# The Evolution and Development of the Antibody Repertoire

**DOI:** 10.3389/fimmu.2015.00033

**Published:** 2015-02-05

**Authors:** Harry W. Schroeder

**Affiliations:** ^1^Department of Medicine, Division of Clinical Immunology and Rheumatology, University of Alabama at Birmingham, Birmingham, AL, USA

**Keywords:** immunoglobulin, antibody repertoire, comparative immunology, developmental immunology, high-throughput sequencing

Approximately 500 million years ago (1), vertebrates developed the ability to generate a highly diverse repertoire of immunoglobulins (Igs). These highly versatile proteins serve as both effector molecules and as receptors for antigen ligands. As soluble effectors, Igs can activate and fix complement and they can bind Fc receptors on the surfaces of granulocytes, monocytes, platelets, and other components of the immune response. V(D)J gene segment rearrangement and somatic hypermutation (SHM) create a population of diverse ligand binding sites that allow recognition of an almost unlimited array of self and non-self antigens. Above and beyond the time-honored practice of vaccination, the power of Igs as biotherapeutic agents is changing the face of medicine. In this research topic, we collected several exciting articles that highlight the diversity and similarity of antibody repertoires. We also highlight new bioinformatics approaches for the analysis of this data.

We open the research topic with a review of antibody repertoires in fish by Fillatreau and colleagues (2). This review provides a description of the organization of fish Ig loci, with a particular emphasis on their heterogeneity between species, and presents recent data on the structure of the expressed Ig repertoire in healthy and infected fish. This is followed by a review of antibody repertoires in pigs (3). In pigs, the fetal repertoire develops without maternal influences and the precocial nature of multiple offspring provides investigators with the opportunity to study the influence of environmental and maternal factors on repertoire development.

Next, we take a closer look at the human repertoire. Vas et al. (4) discuss the role of natural antibodies (Nabs). Mostly, IgM antibodies are produced in the absence of exogenous antigen challenge. The composition of the early immune repertoire is highly enriched for NAbs, which are polyreactive and often autoreactive. Included in Nabs are antibodies that recognize damaged and senescent cells, often via oxidation-associated neo-determinants. Clinical surveys have suggested that anti-apoptotic cell (AC) IgM NAbs may modulate disease activity in some patients with autoimmune disease. This review is followed by a comparative study by Mroczek and colleagues (5) of the antibody repertoire expressed by immature, transitional, mature, memory IgD^+^, memory IgD^−^, and plasmacytes isolated from the blood of a single individual. Differences observed between the Igs produced by these cells indicate that studies designed to correlate repertoire expression with diseases of immune function will likely require deep sequencing of B cells sorted by subset. The next paper highlights secondary mechanisms of antibody diversification that act in addition to V(D)J recombination and SHM of the complementary determining regions (CDRs) of the antibody that create the antigen-binding site (6). These secondary mechanisms include V(DD)J recombination (or D–D fusion), SHM-associated insertions and deletions, and affinity maturation and antigen contact by non-CDR regions of the antibody. Next is an analysis of age-related changes in the antibody repertoire following vaccination by Wu et al. (7). Clustering analysis of high-throughput sequencing data enables us to visualize the response in terms of expansions of clonotypes, changes in CDR-H3 characteristics, and SHM as well as identifying the commonly used IgH genes. This study highlights a number of areas for future consideration in vaccine studies of the elderly.

Finlay and Almagro (8) pull all of these strands together in the final research based article, which reviews the structural studies and fundamental principles that define how antibodies interact with diverse targets. They compare the antibody repertoires and affinity maturation mechanisms of humans, mice, and chickens, as well as the use of novel single-domain antibodies in camelids and sharks. These species utilize a plethora of evolutionary solutions to generate specific and high-affinity antibodies. The various solutions used by these species illustrate the plasticity of natural antibody repertoires. They end their article by discussing man-made antibody repertoires that have been designed and validated in the last two decades. Together, these comparative studies of natural and man-made repertoires served as tools to explore how the size, diversity, and composition of a repertoire impact the antibody discovery process.

High-throughput sequencing is tailor made for the study of antibody repertoires. However, the diversity of the sequences that is obtained from these studies is immense, and thus requires the development of new and friendly bioinformatics techniques to analyze and interpret the data. The final two articles are devoted to methods that can be used for these purposes. The first issue is quality control. Michaeli et al. (9) present a method for automated cleaning and pre-processing of immunoglobulin gene sequences from high-throughput sequencing. Their paper describes Ig high-throughput sequencing cleaner (Ig-HTS-cleaner), a program containing a simple cleaning procedure that successfully deals with pre-processing of Ig sequences derived from HTS, and Ig insertion–deletion identifier (Ig-Indel-identifier), a program for identifying legitimate and artifact insertions and/or deletions (indels). These programs were designed for analyzing Ig gene sequences obtained by 454 sequencing, but they are applicable to all types of sequences and sequencing platforms. Finally, Rogosch et al. (10) present an easy-to-use Microsoft^®^ Excel^®^ based software, named immunoglobulin analysis tool (IgAT), for the summary, interrogation, and further processing of IMGT/HighV-QUEST output files. IgAT generates descriptive statistics and high-quality figures for collections of murine or human Ig heavy or light chain transcripts ranging from 1 to 150,000 sequences. In addition to traditionally studied properties of Ig transcripts – such as the usage of germline gene segments, or the length and composition of the CDR-3 region – IgAT also uses published algorithms to calculate the probability of antigen selection based on somatic mutational patterns, the average hydrophobicity of the antigen-binding sites, and predictable structural properties of the CDR-H3 loop according to Shirai’s H3-rules.

The authors that contributed to this volume hope that the reader will find this research topic interesting, thought-providing, and informative. We invite you to read the following articles and immerse yourself in the fascinating world of Igs. In the near term future, this world is likely to continue to provide new venues for the diagnosis, treatment, or prevention of disease.

## Conflict of Interest Statement

The author declares that the research was conducted in the absence of any commercial or financial relationships that could be construed as a potential conflict of interest.
